# A tribute to our case of the month and a warm welcome to “under your microscope”

**DOI:** 10.1111/bpa.12932

**Published:** 2021-01-28

**Authors:** Caterina Giannini, Clayton Wiley, Markus Glatzel

From its inception, a principal goal of Brain Pathology, as articulated by the founding Editor‐in‐Chief, Dr. Paul Kleihues, was to become the journal of choice for all neuropathologists. Scientific articles were selected from a broad spectrum of submissions with a particular eye toward key progress in the field of neuropathology. While engaging the readership in biomedical advances, we also wanted every issue to have something for every neuropathologist. Beginning with the Dear Reader column, we provided a forum to discuss the current topics in our field and ended with updates from the many national neuropathology societies.

Approximately 25 years ago we added the “Case of the Month” (COM) section as an opportunity for our readership to exchange the classic clinical cases as evaluated by practicing neuropathologists. Meticulously edited by Dr. Ronald Hamilton from its outset, and for the next 25 years, COM received wide acclaim. Beyond being great puzzles, each COM furnished superb diagnostic images for clinical teaching archives. This indeed was Ron’s “baby” which he tirelessly doted over, zealously exchanging emails with contributors to capture the teaching moment of each case. We thank him for this service not only in the name of the editorial board of “Brain Pathology” but also in the name of the global community of diagnostically active neuropathologists.

A quarter of a century later, the information revolution has made the world a very different place. This has impacted diagnostic neuropathology (3), the way we use digital information in neuropathology (1) and the way we teach neuropathology (2), and it is time to update COM. With this issue we are heralding the evolution of COM to “Under Your Microscope.” Taking advantage of virtual microscopy, we are providing our readership with the “real McCoy,” that is, digital microscopy files affording them real‐life challenges of cases as they would appear under their microscope. Readers will no longer be limited to reviewing static captured images, and now have the opportunity to examine a digital documentation of clinical cases. In addition to honing their diagnostic acumen, the digital files will provide an archive of clinical teaching cases. With Caterina Giannini as the section editor for “Under Your Microscope” we entrust this enterprise to a highly competent neuropathologist, who, as the current moderator of the Diagnostic Slide Session of the American Association of Neuropathologists, brings all the credentials necessary to fill this section with life.

As in the past, we are encouraging the submission of cases from the entire spectrum of neurological disease, including diagnostic challenges, rare clinical entities to pathognomonic findings. Online case submission is as easy and straight forward as it for all submissions to Brain Pathology and no Article Publication Charges occur.

After initial editorial review, cases are selected for the submission of either glass slides or for those readers with access to whole slide imaging, submission of the digital slide files. After final editorial review, selected cases will be accepted for the publication in Brain Pathology and posted on our website. Accepted cases will be archived in a database annotated by world‐experts for universal online access. We are excited to see how future generations use this rich teaching and investigational resource.
